# Supplementary material for: A Quartet of Native Orai Channel Isoforms Orchestrates Graded NFAT Activation and Transcription
Source: bioRxiv. 2026 Jul 13:2026.07.10.737753. Preprint. [Version 1] doi: 10.64898/2026.07.10.737753 (PMC13404903; doi:10.64898/2026.07.10.737753)

bioRxiv preprint doi: https://doi.org/10.64898/2026.07.10.737753; this version posted July 13, 2026. The copyright holder for this preprint (which was not certified by peer review) is the author/funder, who has granted bioRxiv a license to display the preprint in perpetuity. It is made available under a CC-BY 4.0 International license.

Supplementary Figure legends

Figure S1. Near-native expression of Orai proteins in Orai-TKO HEK293 cells. (A) Representative fluorescence images of HEK293 Orai-TKO cells expressing either Orai1α, Orai1β, Orai2 or Orai3 using TK promoter-driven expression plasmids. Also shown are images of HEK293 Orai-TKO cells expressing Orai1α and Orai1β when expression is driven by the stronger cytomegalovirus (CMV) promoter. **(B-C)** Comparison of CFP fluorescence intensity in cells transfected with Orai1α-CFP or Orai1β-CFP driven by the TK promotor, showing comparable expression between isoforms. **(D)** Confocal fluorescence images confirming low expression of the four Orai isoforms using TK promoter-driven expression plasmids. Data are expressed as mean ± SEM. Parametric data were analyzed using one-way ANOVA with Dunnett's post hoc test, and nonparametric data were analyzed using the Kruskal–Wallis test with Dunn's multiple comparisons. **(E)** Western blot showing endogenous Orai1 in HEK293 cells, lack of Orai1 expression in HEK293 Orai TKO cells, and expression of Orai1α-CFP or Orai1β-CFP following their reconstitution in the HEK293 TKO background. Orai1α-CFP and Orai1β-CFP migrate at higher molecular weights than endogenous Orai1 due to CFP tagging, with Orai1α-CFP appearing above Orai1β-CFP, consistent with its longer N-terminus. HSP70 was used as a loading control.

Figure S2. Endogenous expression levels of Orai1α and Orai1β across polarized CD4$^+$ T-cell subsets. (A) Naïve CD4$^+$ T cells were isolated from mouse spleen and lymph nodes by negative selection and activated with anti-CD3/CD28 under lineage-skewing conditions to generate Th1, Th2, iTreg, cTh17, and pTh17 cells. **(B)** Orai1α and Orai1β protein expression was assessed by Western blotting in naïve and polarized CD4$^+$ T-cell subsets. **(C)** Quantification shows the relative Orai1β/Orai1α expression ratios across conditions.

Figure S3. High level of expression of Orai1α or Orai1β suppresses SOCE. (A) Retroviral transduction of CD4$^+$ T cells from *Orai1*$^{fl/fl}$*Cd4*^{Cre} mice with Orai1α-IRES-Ametrine, Orai1β-IRES-Ametrine or empty vector (EV) containing the Ametrine reporter. **(B)** Representative flow cytometry plots showing ectopic Orai1 expression (APC) in transduced (Ametrine$^+$) CD4$^+$ T cells. Fixed and permeabilized cells were stained with a C-terminal anti-Orai1 antibody. **(C)** Gating strategy used for SOCE measurements in CD4$^+$ T cells in E and Figure 5A. Live, CD4$^+$ T cells were gated on Ametrine^{high} (top 25th percentile) or Ametrine^{low} (bottom 25th percentile) populations. **(D)** Mean fluorescence intensity (MFI) of Orai1 expression in Ametrine^{low} T cells (as in panel C) transduced with Orai1α, Orai1β, or EV. Fixed and permeabilized cells were stained with a C-terminal anti-Orai1 antibody. **(E)** SOCE measured in Ametrine^{high} CD4$^+$ T cells transduced with Orai1α, Orai1β, or EV and loaded with Indo-1. Cells were stimulated with thapsigargin (TG) in Ca2$^+$ free buffer followed by readdition of 1 mM Ca^{2+} and analyzed by flow cytometry. Data are from 11 mice and four repeat experiments with one technical replicate per condition. Statistical analysis in panels (**D, E**) by ordinary one-way ANOVA multiple comparison. All results are expressed as means ± SEM. ns, not significant ($P > 0.05$).

bioRxiv preprint doi: https://doi.org/10.64898/2026.07.10.737753; this version posted July 13, 2026. The copyright holder for this preprint (which was not certified by peer review) is the author/funder, who has granted bioRxiv a license to display the preprint in perpetuity. It is made available under a CC-BY 4.0 International license.

Figure S4. STIM1 clusters in T cells from wildtype and Orai1α null heterozygote and homozygote individuals. (A) STIM1 clusters in T cells derived from WT, heterozygous and homozygous individuals for the Orai1α null variant (P43T). Top row: Airy Scan images of the clusters induced by thapsigargin were analyzed using 3D Spot Segmentation and the volume of each cluster measured. Bottom row: typical examples of the cluster pattern at the bottom of the cells recorded in the three genotypes. **(B)** Summary of the average cluster volume plotted on a per cell basis in the three genotypes. Data are shown as Mean ± SEM with statistical significance assessed using one-way ANOVA followed by Dunnett post-hoc test.

Figure S5. The Orai1α -specific N-terminus has low evolutionary conservation. (A, B) Phylogenetic tree **(A)** and multiple sequence alignment **(B)** showing the sequence similarity of the Orai1α-specific N-terminus across different species. Sequences were aligned using UniProt alignment tools. Conserved residues are highlighted. **(C)** Percent identity matrix showing pairwise sequence identities for the Orai1α-specific N-terminus across different species.

Supplementary Table

Table S1. Antibodies used for flow cytometric analysis of murine T cells.

Mouse Antigen	Clone	Conjugated fluorochrome	Source	Cat#	RRID	Dilution
CD4	GK1.5	APC/Cy7	BioLegend	100413	AB_312698	1:500
CD4	GK1.5	PE/Cy7	BioLegend	100422	AB_312707	1:500
IFN-γ	XMG1.2	PE	BioLegend	505807	AB_315401	1:200
TNF-α	MP6-XT22	APC	eBioscience	17-7321-82	AB_469508	1:200
IL-2	JES6-5H4	FITC	eBioscience	11-7021-82	AB_465382	1:100
Anti-Orai1	N/A	N/A	YenZym	YZ6856	N/A	1:100
Goat anti-rabbit IgG	N/A	Alexa Fluor 647	Invitrogen	A21244	AB_2535812	1:2000
Indo-1	N/A		BD Biosciences	553258	AB_394738	1: 500
Live/Dead	N/A	Blue	Thermo Fisher	L23105	AB_3717566	1:500

bioRxiv preprint doi: https://doi.org/10.64898/2026.07.10.737753; this version posted July 13, 2026. The copyright holder for this preprint (which was not certified by peer review) is the author/funder, who has granted bioRxiv a license to display the preprint in perpetuity. It is made available under a CC-BY 4.0 International license.

Supplementary Figure 1

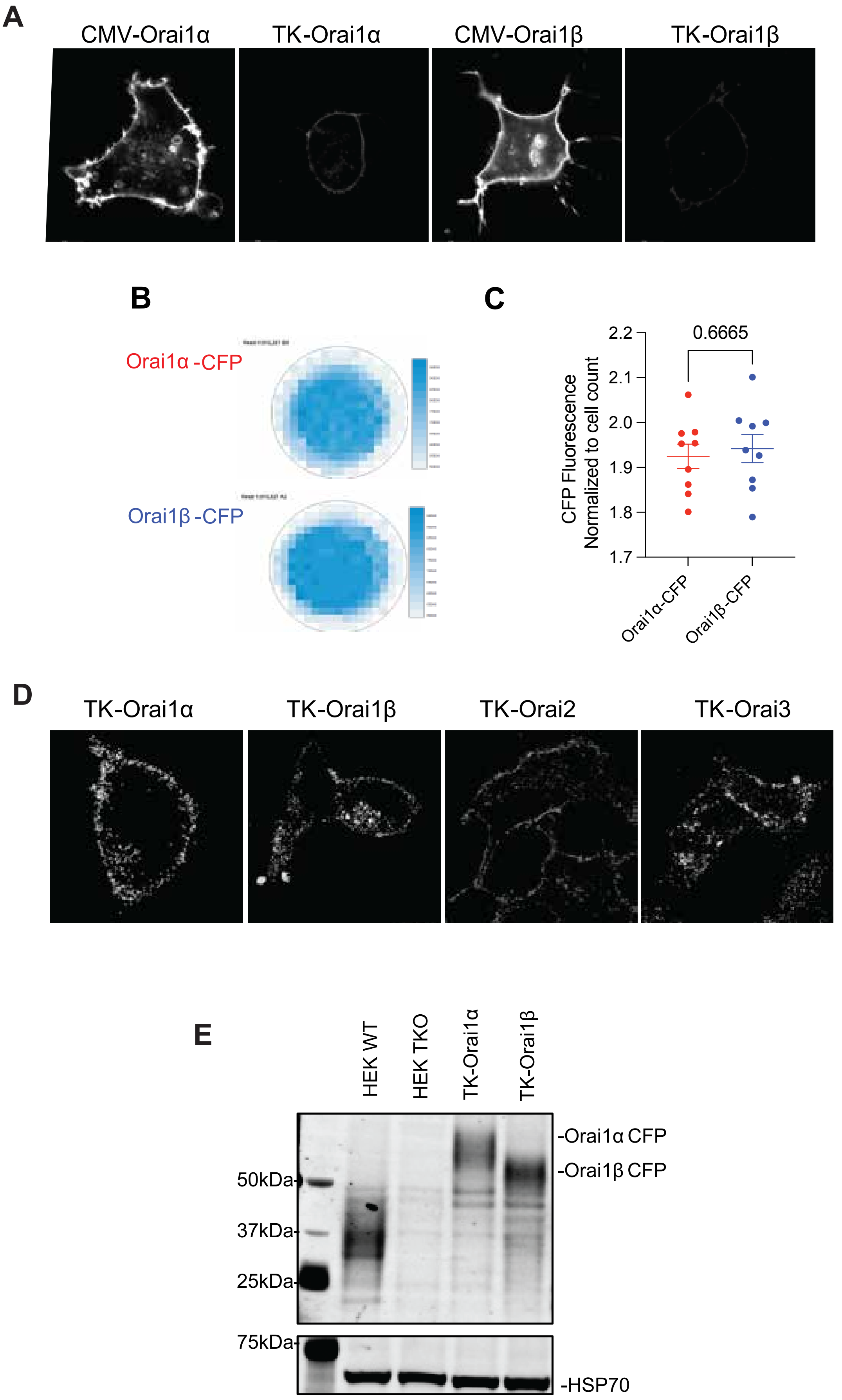

bioRxiv preprint doi: https://doi.org/10.64898/2026.07.10.737753; this version posted July 13, 2026. The copyright holder for this preprint (which was not certified by peer review) is the author/funder, who has granted bioRxiv a license to display the preprint in perpetuity. It is made available under a CC-BY 4.0 International license.

Supplementary Figure 2

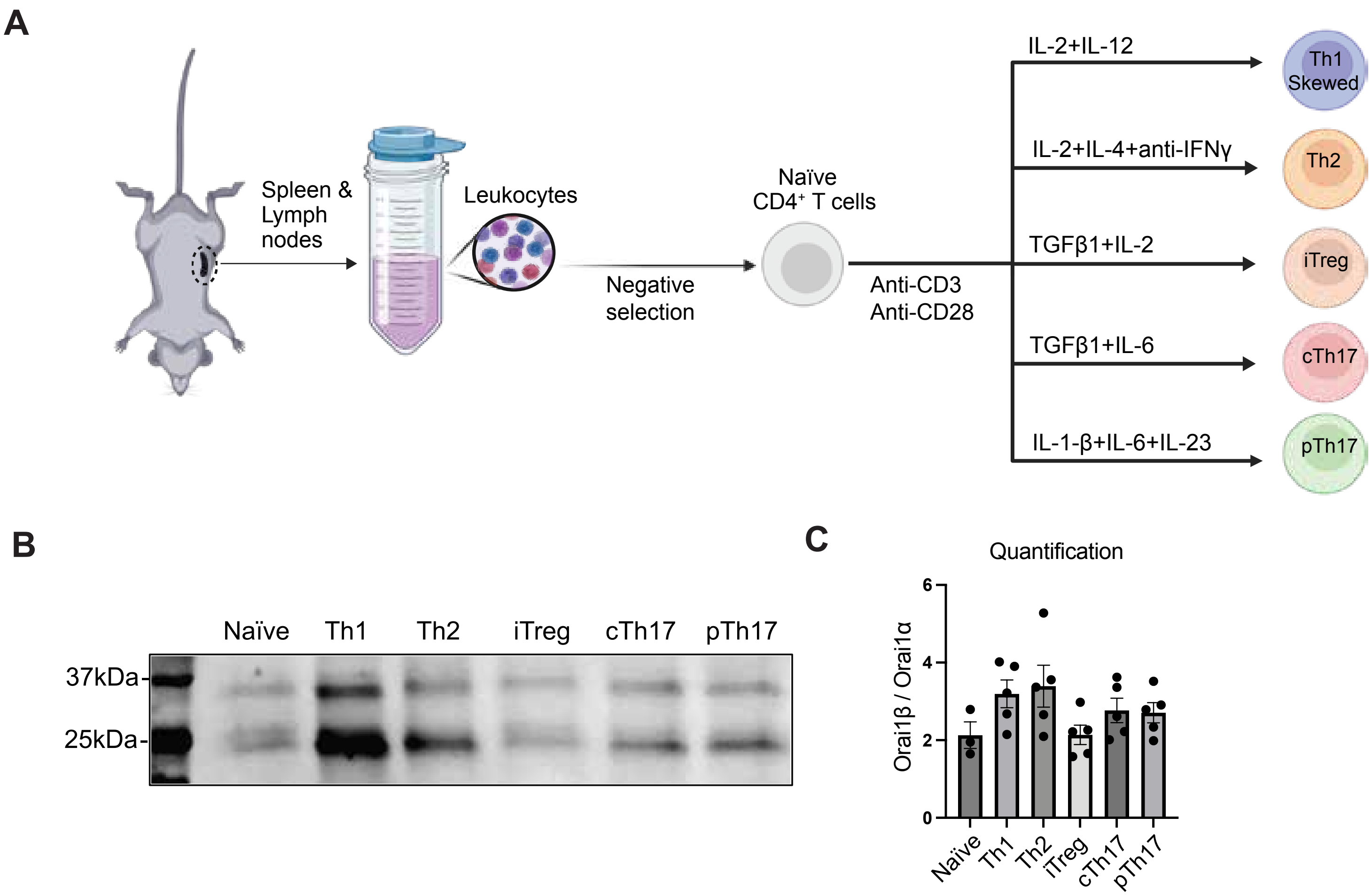

bioRxiv preprint doi: https://doi.org/10.64898/2026.07.10.737753; this version posted July 13, 2026. The copyright holder for this preprint (which was not certified by peer review) is the author/funder, who has granted bioRxiv a license to display the preprint in perpetuity. It is made available under a CC-BY 4.0 International license.

Supplementary Figure 3

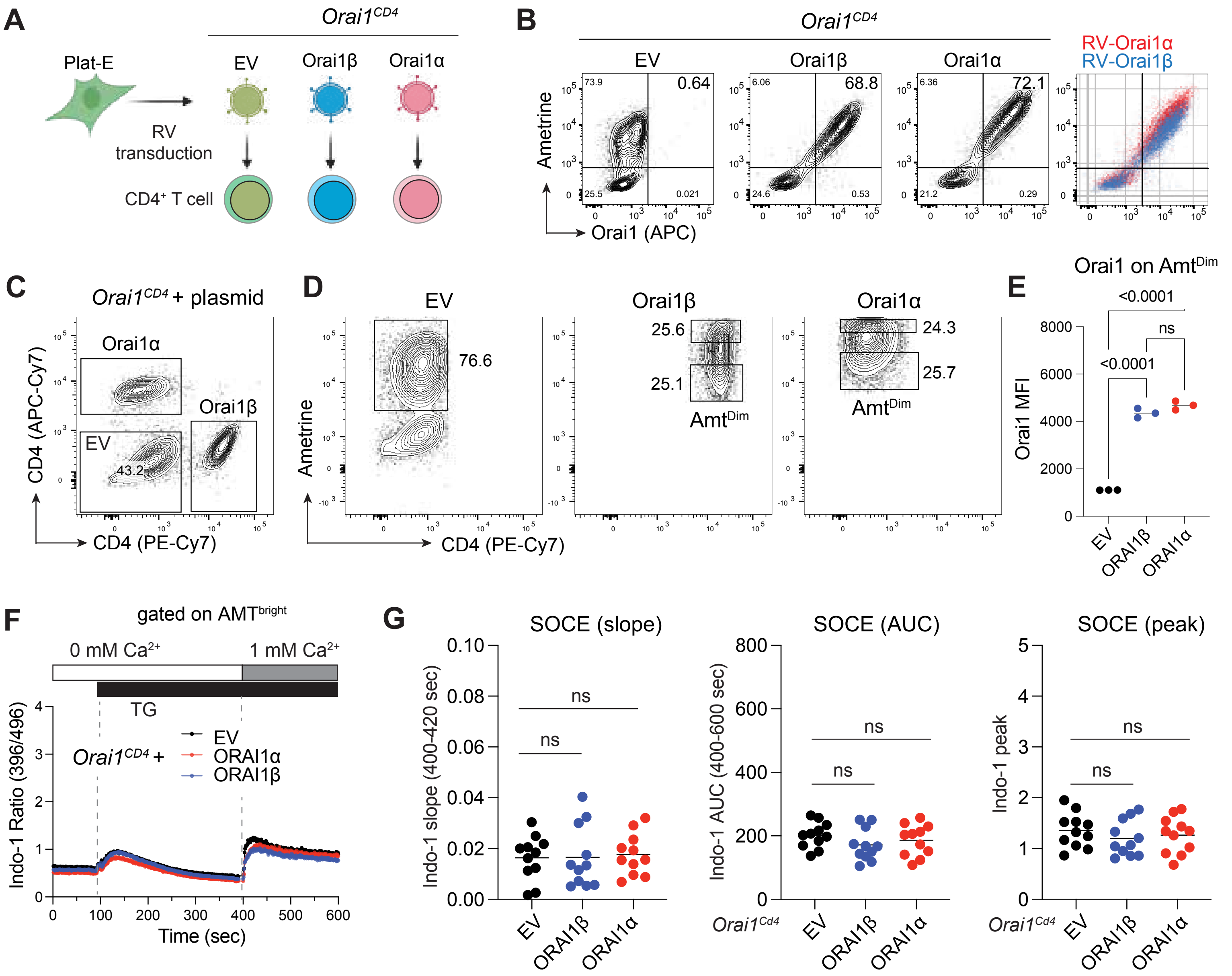

bioRxiv preprint doi: https://doi.org/10.64898/2026.07.10.737753; this version posted July 13, 2026. The copyright holder for this preprint (which was not certified by peer review) is the author/funder, who has granted bioRxiv a license to display the preprint in perpetuity. It is made available under a CC-BY 4.0 International license.

Supplementary Figure 4

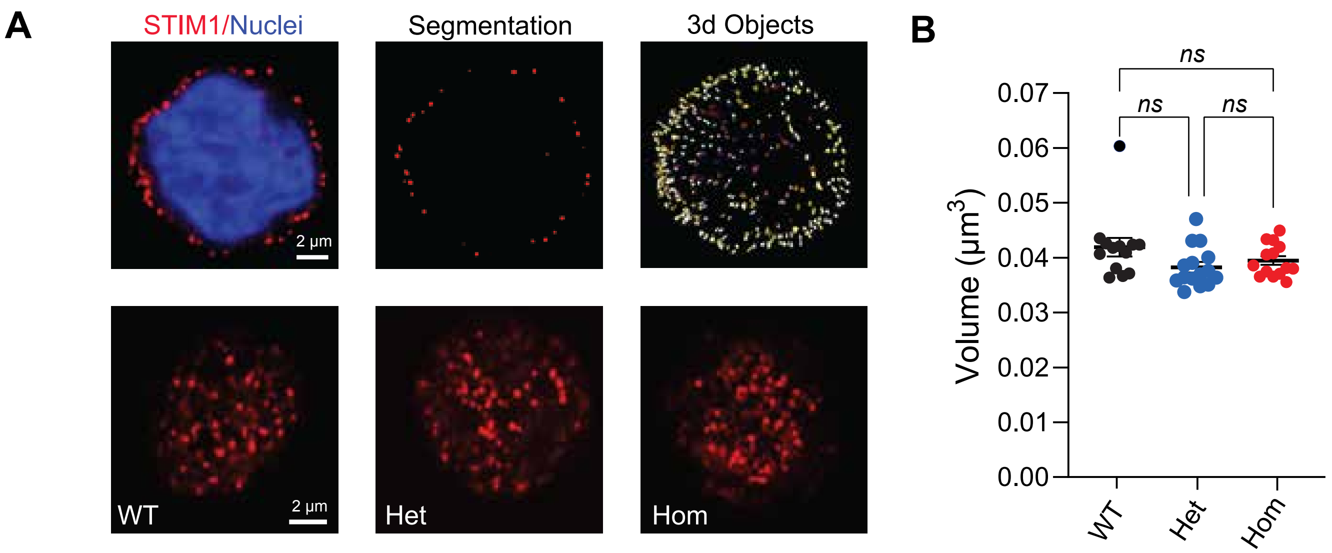

bioRxiv preprint doi: https://doi.org/10.64898/2026.07.10.737753; this version posted July 13, 2026. The copyright holder for this preprint (which was not certified by peer review) is the author/funder, who has granted bioRxiv a license to display the preprint in perpetuity. It is made available under a CC-BY 4.0 International license.

Supplementary Figure 5

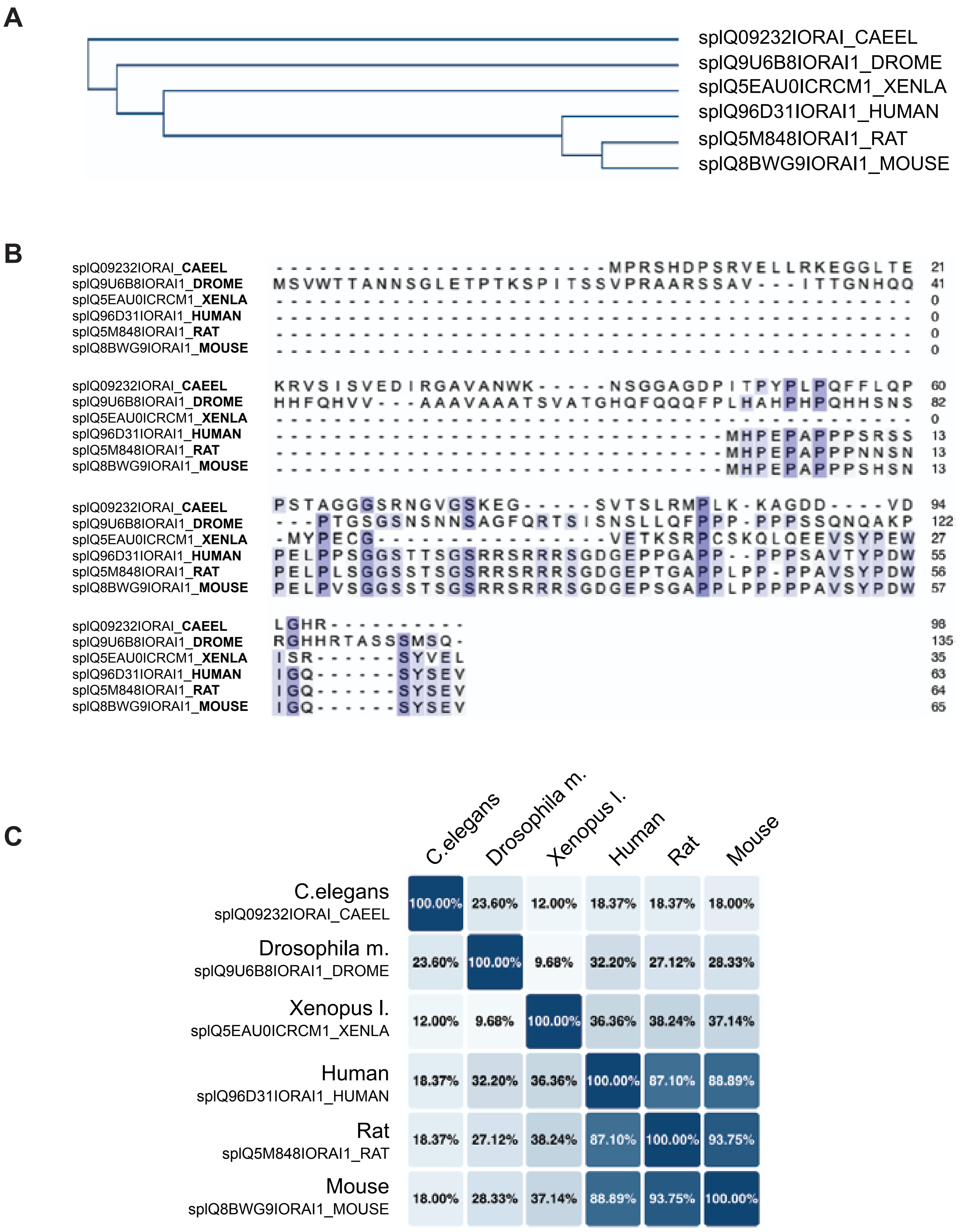

Supplement: Supplement 1 [file NIHPP2026.07.10.737753v1-supplement-1.pdf]
